# Psychosis with paranoid delusions after a therapeutic dose of mefloquine: a case report

**DOI:** 10.1186/1475-2875-5-74

**Published:** 2006-08-23

**Authors:** Tuan M Tran, Joseph Browning, Mary L Dell

**Affiliations:** 1 Emory University School of Medicine, Emory University, Atlanta GA 30322, USA; 2 Department of Psychiatry, Emory University School of Medicine, Atlanta, GA 30322, USA

## Abstract

**Background:**

Convenient once-a-week dosing has made mefloquine a popular choice as malaria prophylaxis for travel to countries with chloroquine-resistant malaria. However, the increased use of mefloquine over the past decade has resulted in reports of rare, but severe, neuropsychiatric adverse reactions, such as anxiety, depression, hallucinations and psychosis. A direct causality between mefloquine and severe reactions among travelers has been partly confounded by factors associated with foreign travel and, in the case of therapeutic doses of mefloquine, the central nervous system manifestations of *Plasmodium *infection itself. The present case provides a unique natural history of mefloquine-induced neuropsychiatric toxicity and revisits its dose-dependent nature.

**Case presentation:**

This report describes an acute exacerbation of neuropsychiatric symptoms after an unwarranted therapeutic dose (1250 mg) of mefloquine in a 37-year-old male previously on a once-a-week prophylactic regimen. Neuropsychiatric symptoms began as dizziness and insomnia of several days duration, which was followed by one week of escalating anxiety and subtle alterations in behaviour. The patient's anxiety culminated into a panic episode with profound sympathetic activation. One week later, he was hospitalized after developing frank psychosis with psychomotor agitation and paranoid delusions. His psychosis remitted with low-dose quetiapine.

**Conclusion:**

This report suggests that an overt mefloquine-induced psychosis can be preceded by a prodromal phase of moderate symptoms such as dizziness, insomnia, and generalized anxiety. It is important that physicians advise patients taking mefloquine prophylaxis and their relatives to recognize such symptoms, especially when they are accompanied by abrupt, but subtle, changes in behaviour. Patients with a history of psychiatric illness, however minor, may be at increased risk for a mefloquine-induced neuropsychiatric toxicity. Physicians must explicitly caution patients not to self-medicate with a therapeutic course of mefloquine when a malaria diagnosis has not been confirmed.

## Background

Mefloquine, a 4-quinoline methanol structurally related to quinine, has a long elimination half-life of 14 to 41 days [1], which allows for once-a-week dosing and makes it a popular choice as malaria prophylaxis for travelers to malaria endemic areas. Along with atovaquone/proguanil and doxycycline, mefloquine is currently recommended as malaria prophylaxis for travel in areas with chloroquine-resistant *Plasmodium falciparum *by health authorities in the United States, the United Kingdom, and Canada [2,3]. The current recommended prophylactic dose is 250 mg once a week for adults. Since mefloquine was introduced in 1985, the most commonly reported adverse events have been gastrointestinal and neuropsychiatric events [4,5]. It is the latter that has been the most concerning to the public. Common neuropsychiatric adverse events include dizziness, insomnia, and strange or vivid dreams. The increased use of mefloquine over the past two decades, due to increased international travel to malaria endemic areas, has resulted in reports of less common, but more severe, neuropsychiatric reactions such as anxiety, depression, hallucinations and psychosis. Due to the relative rarity of events, the causal relationship between mefloquine and these severe reactions among travelers is tenuous, often confounded by factors associated with foreign travel and, in the case of therapeutic doses of mefloquine, the central nervous system (CNS) manifestations of *Plasmodium *infection itself.

This report describes an acute exacerbation of neuropsychiatric symptoms after an unwarranted therapeutic dose (1250 mg) of mefloquine in a 37-year-old male previously on a once-a-week prophylactic regimen. The case demonstrates the time course of mefloquine-induced neuropsychiatric toxicity, revisits the dose dependent nature of such adverse events, and reviews the current literature.

## Case presentation

Mr. A, a 37-year-old Nigerian male who has lived in the United States for ten years, traveled to Nigeria for one week with his wife to visit family. He had never taken mefloquine prophylaxis before this trip, but his childhood was remarkable for multiple episodes of malaria treated with various anti-malarial regimens. His previous psychiatric history was significant for a single panic attack experienced in his early twenties. He had no medical diagnoses prior to these events. He used no alcohol or other substances and had no known drug allergies.

Mr. A began his malaria prophylaxis regimen of once-a-week, 250 mg mefloquine two weeks prior to traveling to Nigeria. He and his wife spent a week in urban regions of his native country without incident. He discontinued his malaria prophylaxis upon returning to the United States. Two weeks later, Mr. A began experiencing fatigue and a mild headache and presented to a university-affiliated travel clinic. He denied fever, chills, night sweats, or nausea. Chemistries and cell blood count were within normal limits, and a blood smear showed no evidence of parasitaemia.

Two weeks after his visit to the travel clinic, Mr. A, still concerned that he might have malaria, took a one-time therapeutic dose of 1250 mg mefloquine. The next day, he began experiencing vertigo and insomnia, both which lasted for several days. While at work that same week, he experienced chest pain radiating down his left arm with concomitant diaphoresis and tachycardia in what the patient described as "an ACS picture," referring to acute coronary syndrome. He felt intense anxiety at this time. Mr. A's blood pressure taken in his office was 210/110. He then went to the emergency department of an outside hospital and was admitted for a hypertensive emergency. Cardiac work-up for ACS, including an exercise stress test, was negative and head computerized tomography showed no abnormalities other than a left maxillary mucosal inclusion cyst suggestive of sinusitis. Mr. A was diagnosed with hypertension and, incidentally, with Type II diabetes mellitus. He was held overnight for observation and discharged the following morning on metoprolol 100 mg qd, lisinopril 10 mg qd, rosiglitazone 1 mg bid, and metformin 500 mg bid, and zolpidem 10 mg qhs. The following Monday morning, he returned to work at his office, but left midday because he was experiencing anxiety and nervousness. He did not return to work the rest of the week and tried to relieve his anxiety with exercise and outdoor activities. During this week, he began having unusual conversations about spirituality and religion with his wife. His wife reported that he had difficulty following conversations and was remarkably suspicious of her as well, with numerous inquiries into her activities and friends.

Nine days after the previous hospital discharge, Mr. A became abruptly anxious and agitated with delusions that his wife was having an extramarital affair and began "talking out of his head." His wife, fearful of his increasing agitation and paranoia, finally coaxed Mr. A into going to the emergency department at a university-affiliated hospital. He became severely agitated and paranoid in the emergency department, becoming highly suspicious of the one-to-one sitter ordered for his safety after he had reported suicidal ideation. He attempted to escape from the hospital. Eventually, Mr. A had to be restrained to his bed due to profound psychomotor agitation. His vital signs were remarkable for a blood pressure of 157/97, and physical examination was significant only for altered mental status. Serum electrolytes, renal function, and cell blood count were within normal limits. Blood glucose was mildly elevated at 146 mg/dL. Urine drug screen was negative. Urinalysis was positive for bacteriuria, and a course of levofloxacin was initiated. Mr. A was admitted to the general medicine service, where overnight he had another acute episode of agitation, becoming increasingly argumentative with the staff. Intramuscular haloperidol was administered to treat his acute psychotic agitation.

Mr. A was transferred to the medical psychiatry inpatient unit with the DSM-IV-TR diagnosis of Psychotic Disorder, Not Otherwise Specified. His vital signs remained stable. Rapid plasma reagin test, cobalamin and folate levels, liver transaminase levels, and thyroid stimulating hormone levels were within normal limits. Mr. A complained of intense anxiety with no identifiable precipitant on the first day of his hospital course. He demonstrated paranoid behaviour and was highly suspicious of the staff, frequently checking identification badges and quizzing the staff in order to verify their background and knowledge. Although generally cooperative, he questioned the utility of all procedures indicated in his treatment. He was started on quetiapine 25 mg qhs, alprazolam 0.5 mg bid and zolpidem 5 mg qhs. The latter two medicines were both started during his previous hospitalization for hypertensive crisis. He was also maintained on his previous anti-hypertensive (metoprolol 100 mg qd and lisinopril 10 mg qd) and diabetic (rosiglitazone 1 mg bid, and metformin 500 mg bid) regimens. An electroencephalogram was performed, and the results were negative and inconsistent with delirium or encephalopathy. His vital signs were stable and within normal limits.

The next day, he reported that he refused quetiapine the previous night (confirmed with the nursing staff), citing that his paranoia and delusions had resolved. His claims were incongruent with his behaviour, as he was even more inquisitive than previously, again challenging the staff on their medical knowledge and questioning the rationale behind his medical treatment. He requested that he see his medications delivered in their original packaging before taking them. He eventually took his psychotropic medications but refused diabetic management on grounds that he wanted a subspecialist to manage his diabetes. Work-up for pheochromocytoma, porphyria, and heavy metal toxicity was negative. Over the next couple days, Mr. A's paranoia remitted in response to low dose quetiapine, and he became more pleasant, agreeable, and less suspicious. He eventually agreed to the nursing staff dispensing all his medications and to routine management of his diabetes. On day six of admission, Mr. A stated that he felt more trusting and was completely agreeable to the treatment and discharge plan, although he still found it difficult to follow conversations. After confirming follow-up appointments with Mr. A and his wife and obtaining assurances that he would not return to work until after clearance by an outpatient psychiatrist, Mr. A was discharged on quetiapine 100 mg qhs, metoprolol 100 mg qd, lisinopril 10 mg qd, rosiglitazone 1 mg bid, and metformin 500 mg bid.

## Conclusion

Mr. A's initial presenting symptoms in the travel clinic were fatigue and a mild headache, both non-specific symptoms that may or may not be attributable to mefloquine. One can assume that Mr. A tolerated the prophylactic mefloquine regimen relatively well as evidenced by the absence of serious neuropsychiatric symptoms during and immediately after his three-week course. However, these lingering non-specific symptoms prompted him to take a therapeutic dose of mefloquine despite a negative blood smear. His neuropsychiatric symptoms appeared the next day, concomitant with the presumed peak of mefloquine, which reaches maximum concentration in the blood within 24 hours of an oral dose [1].

Mr. A's risk factors for mefloquine-induced psychosis include previous use of anti-malarials for childhood malaria and a single panic attack that occurred ten years prior to this psychotic episode. Drug interactions between mefloquine and anti-hypertensive or anti-diabetic medications may have contributed to the psychosis, but this is unlikely given that Mr. A experienced sleep disturbances, vertigo, anxiety and a panic attack prior to being diagnosed with diabetes and hypertension. Current literature lacks evidence of such interactions aside from one study which demonstrated that mefloquine inhibited the metabolism of metoprolol [6]. Similarly, the contribution of incidental bacteriuria to Mr. A's altered mental status is plausible but unlikely, given that he had neither pre-existing cognitive impairment nor clinical findings that would suggest a complicated urinary tract infection. Levofloxacin was used to treat his bacteriuria after the initial onset of neuropsychiatric symptoms. Interestingly, levofloxacin is a fluoroquinolone antibiotic structurally related to mefloquine but has a low overall rate of serious CNS adverse events, with such events occurring in only 4 per 100 000 treated patients [7]. Whether levofloxacin can contribute to pre-existing neuropsychiatric symptoms in an additive manner has yet to be formally investigated. Mr. A's psychotropic medications should not have contributed significantly to his psychotic presentation. Both alprazolam and zolpidem are short-acting drugs that were well tolerated at low doses for one week prior to the acute psychotic episode. In addition, alprazolam, as are other benzodiazepines, has an insignificant risk of inducing psychosis; in fact, this class of drugs has been used in the treatment of acute psychosis [8-10]. It is important to note that zolpidem has been reported to induce psychosis at much higher doses, but this phenomenon has been limited to women [11].

A review of neuropsychiatric adverse events in an estimated 1.2 million mefloquine prophylaxis users from continental Europe demonstrated only 278 adverse events (0.023%), with the most commonly reported being dizziness, neuropathies, headaches, and psychiatric disorders of affect [12]. The study was limited by its reliance on self-reporting of adverse events and the use of a crude, overestimated denominator derived from drug sales rather than actual compliance rates. More recent studies have consistently demonstrated that neuropsychiatric adverse events associated with prophylaxis are usually limited to dizziness, bizarre or vivid dreams, and insomnia, but with incidence rates of 10–40% [4,5,13]. Anxiety is less common, with one trial (n = 483) reporting an incidence of 4% in non-immune travelers receiving mefloquine prophylaxis [4]. Mefloquine-induced panic attacks and psychosis appear to be even rarer events; an observational study of 16,491 subjects on mefloquine prophylaxis described only 9 and 3 events, respectively, during the course of mefloquine use [14].

Neuropsychiatric adverse events after *therapeutic *doses of mefloquine have been difficult to study mainly because malaria itself often causes CNS symptoms, and current practices in endemic regions require mefloquine/artesunate combination therapy due to increasing mefloquine resistance [15]. Nevertheless, in a limited study of 22 healthy volunteers, almost all participants experienced vertigo, nausea, or headache after a therapeutic dose of mefloquine. Less common events included anxiety, depression, confusion and hallucinations [16]. An earlier risk analysis of malaria cases in Germany suggested that therapeutic mefloquine increased the risk of neuropsychiatric adverse events by 60-fold over prophylactic doses [17]. In the treatment of uncomplicated falciparum malaria with mefloquine regimens, randomized, controlled trials in Thailand have consistently shown dose-related increases in both moderate and severe neuropsychiatric adverse events [18-21]. Pronounced CNS toxicity was seen in two cases of inadvertent mefloquine overdose secondary to dispensing errors [22]. One patient developed confusion, agitation, ataxia, dizziness, speech difficulties, and high-frequency hearing loss after taking 15 250 mg of mefloquine over two months. The other patient complained of persistent weakness, depression, disorientation, and paresthesia after taking 16 250 mg of mefloquine within a six month period. In both cases, mefloquine (Lariam) was mistakenly substituted for terbinafine (Lamisil). These data suggest that the incidence and severity of neuropsychiatric events associated with mefloquine use is dose-dependent. Interestingly, serious adverse events do not seem to correlate with mefloquine levels in the blood [23], implying that the risk of developing neuropsychiatric symptoms may be related to an individual's threshold for mefloquine rather than absolute mefloquine blood levels. The case presented here supports a dose-dependent effect of mefloquine neurotoxicity, as Mr. A developed profound anxiety and subsequent paranoid psychosis only after his dose was escalated from 250 mg to a one-time dose of 1250 mg.

To date, the effects of mefloquine on the CNS remain unclear, given the limited literature on this topic. On a biochemical level, there is evidence that mefloquine may disrupt calcium homeostasis and induce a stress response in rat neurons *in vitro *[24]. Also, mefloquine has been shown to inhibit electrical coupling between interneurons in slices of rat neocortex via blockade of gap junctional channels [25]. At the clinical level, Croft and Herxheimer critically reviewed 516 case reports and proposed that mefloquine exerts its CNS effects via primary hepatotoxicity and symptomatic thyroid dysfunction [26]. In the present report, work up for hepatic and thyroid dysfunction was negative.

This case reveals a unique and insidious natural history of mefloquine neurotoxicity. Prior to developing overt psychosis, Mr. A had initially experienced vertigo and insomnia for several days followed by a full week of escalating anxiety and subtle alterations in behaviour. His anxiety culminated into a panic episode with profound sympathetic activation within a week of taking therapeutic mefloquine. This suggests that an overt mefloquine-induced psychosis can be preceded by a prodromal phase of moderate symptoms such as dizziness, insomnia, and generalized anxiety. Taylor and White reviewed several studies assessing the effect of mefloquine on psychomotor performance and fine motor skills and concluded that mefloquine did not significantly interfere with higher mental function despite causing adverse effects such as sleep disturbances and changes in affect [20]. The fact that mefloquine toxicity does not usually hinder daily function may mask its potential severity. It is important that physicians advise patients taking mefloquine and their relatives to recognize specific symptoms, especially when they are accompanied by abrupt but subtle changes in behaviour.

Although psychosis is usually self-limited after mefloquine is discontinued, its adverse effects on the CNS may continue for several weeks due to a long elimination half-life. Therapy with atypical antipsychotics, such as risperidone, has shown to be effective in several cases, with psychosis remitting within a few days [27-29]. Mr. A responded within two days to low-dose quetiapine with complete resolution of his psychomotor agitation and paranoid delusions. Therefore, recognition of specific neuropsychiatric symptoms and behavioural changes in a patient taking mefloquine may prompt physicians to more carefully assess the patient and intervene if necessary before severe psychosis develops.

## Authors' contributions

TT, JB, and MD were involved in the management and care of the patient. TT prepared the manuscript with contribution from MD. All authors read and approved the final manuscript.
